# Dynamical Mechanism of Polarons and Bipolarons in Poly(*p*-Phenylene Vinylene)

**DOI:** 10.1038/s41598-019-54748-0

**Published:** 2019-12-02

**Authors:** Fábio Luís de Oliveira Paula, Leonardo Luiz e Castro, Luiz Antonio Ribeiro Junior, Rafael Timóteo de Sousa Júnior, Geraldo Magela e Silva, Pedro Henrique de Oliveira Neto

**Affiliations:** 1 Institute of Physics, University of Brasília, Brasília, 70910-900 Brasília, Brazil; 2 University of Brasília, PPG-CIMA, Campus Planaltina, 73345-010 Brasília, DF Brazil; 3 Department of Electrical Engineering, University of Brasília, CP04455, Brasília, 70919-970 Brazil

**Keywords:** Materials science, Condensed-matter physics, Materials for devices, Materials for optics, Theory and computation

## Abstract

Studies on Poly(p-Phenylene Vinylene) (PPV) and derivatives have experienced enormous growth since they were successfully used to fabricate the first efficient prototypes of Polymer Light-Emitting Diodes in the 90s. Despite this rapid progress, understanding the relationship between charge transport and the morphology in these materials remains a challenge. Here, we shed light on the understanding of the transport mechanism of polarons and bipolarons in PPVs by developing a two-dimensional tight-binding approach that includes lattice relaxation effects. Remarkably, the results show that the PPV lattice loses the energy related to its conjugation during time by transferring this amount of energy to electrons. Such a process for energy transfer permits the quasiparticles to overcome the potential barrier imposed by the local lattice deformations, that are formed in the presence of an additional charge and, consequently, their electric field assisted transport takes place. Within the framework of this transport mechanism, a better insight into the origin of the carrier mobility in PPV and derivatives can be achieved and would be a useful guide for improving their chemical structures and morphologies.

## Introduction

Polymer Light-Emitting Diodes (PLEDs) are currently considered the most prominent candidates for developing new display technologies with good cost-efficiency compromise^1,2^. The awakening of interest for this field of research have initiated in the discovery of the Poly(p-Phenylene Vinylene) (PPV) light-emitting properties that are a result of both easy processing and mechanical flexibility presented by this material^3^. In a PLED, electrons and holes are injected into the polymer layer forming self-localized quasiparticles due to the strong mutual interaction between charge and lattice deformations, namely polarons. This carrier species has spin ±1/2 and charge ±*e*^4^. Alternatively, bipolarons are generated in PLEDs when the process of injecting charge results in a large concentration of polarons. In this sense, two acoustic polarons with the same charge and antiparallel spins can merge to a bipolaron, that is a spinless charge carrier with charge ±2*e*^4^. Recognizably, polarons and bipolarons are the primary structures in playing the role of the charge transport mechanism in conjugated polymers^5^. They are composite states in which their stability strongly depends on the lattice degrees of freedom. Therefore, determining the relationship between charge transport and morphology is crucial to increasing the charge carrier mobility of PPVs. However, the impact of these features interplay on the PLED performance is still unclear.

So far, the majority of investigations to understand the nature of the charge transport mechanism in PPVs have focused on experiments of time-of-flight (TOF) measurements involving PLEDs and field-effect transistors^6–15^. Importantly, some studies have also measured the mobility of charge carriers along isolated PPV chains^16^. By using the TOF technique, Blom and coworkers have examined the hole transport in PPVs and derivatives as a function of electric field, temperature, frequency, and layer thickness^6–9,17^. Their main results show that the dispersive transport of holes governs the charge transport mechanism in these materials. The dispersion in the hole transport is due to mainly structural disorder, rather than to energetic disorder. Moreover, their findings also demonstrate that an enhancement in the space-charge-limited hole current of PPV derivatives may take place at high bias and room temperature due to the carrier density dependence of the hole mobility. As a counterpoint, signatures of non-dispersive hole transport in soluble PPV derivatives were found by also employing TOF measurements^18^. The results indicate that transport properties depend on both the chemical structures of the polymers and solvents used for film preparation. Depending on the solvent used, PPV films can exhibit either non-dispersive or dispersive transport. Interestingly, these results open channels for controlling the performance of PPV-based devices through careful selection of solvents. From the theoretical standing point, the effect of the interplay between torsional disorder of structural units and the charge transport in PPVs was studied by using a one-dimensional tight-binding approximation^19,20^. The results reveal that static or dynamic ring torsions only impose temporary restrictions of localization on propagating charge carriers^19^. Moreover, other theoretical studies have also shown that PPV chains are much more planar structures regarding other polymer species, such as polythiophene^20^. Since torsion effects have a small impact on the overall carrier dynamics in PPVs, it is essential to elucidating other vital mechanisms that establish the nature of the charge transport in these materials.

In the present work, we investigate the transport mechanism of polarons and bipolarons in PPVs using a 2D tight-binding Hamiltonian that considers lattice relaxation effects. Our approach defines a set of parameters that model a PPV lattice semi-empirically. The central finding obtained here shows that the loss of conjugation in the lattice activates the transport of charge carriers at isolated chains of this material. This result suggests that charge transport can be improved by tuning the effective conjugation along the PPV chain.

## Results

**Figure 1:**
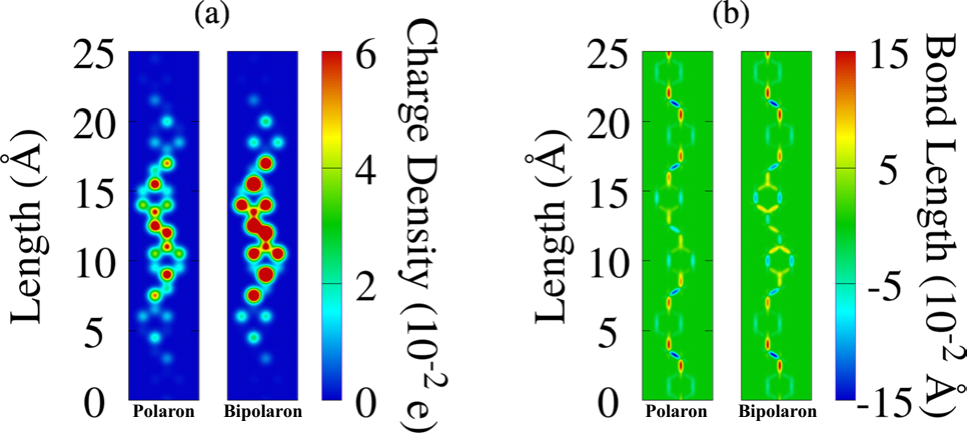
(**a**) Mean charge density and (**b**) bond-length for a polaron (left strip) and a bipolaron (right strip) in the ground state arrangement.

We begin the discussions of our results by presenting the features of charge localization (Fig. 1(a)) as well as related lattices distortions (Fig. 1(b)) for a PPV chain endowed of a polaron and a bipolaron. The left strip in both panels denote the charge and bond-length patterns for a lattice containing a polaron whereas the right strips are referring to the bipolaron case. For the sake of clarity, we show the region in the chain where the charge is localized. In Fig. 1(a) one can note that the charge localization similarly takes place for both charge carriers, in which most of the charge lies in two PPV units. Since bipolarons have twice as much charge than polarons, one can realize stronger signatures of hot colors in the right strip of Fig. 1(a), denoting the formation of a stable bipolaron. In this doubly charged bound state, two polarons merge to form a bipolaron by the overlap of a common lattice distortion, which enhances geometrical relaxation of the bond lengths as illustrates the right strip of Fig. 1(b). Moreover, one can note that the formation of polarons and bipolarons alters the conjugation pattern, *i*.*e*. the alternation between *σ* (red) and *π* (blue) carbon-carbon bonds as presented in regions with the absence of charge. These signatures for charge localization and local lattice deformations are pieces of evidence that a self-interacting state was formed involving both components.

**Figure 2:**
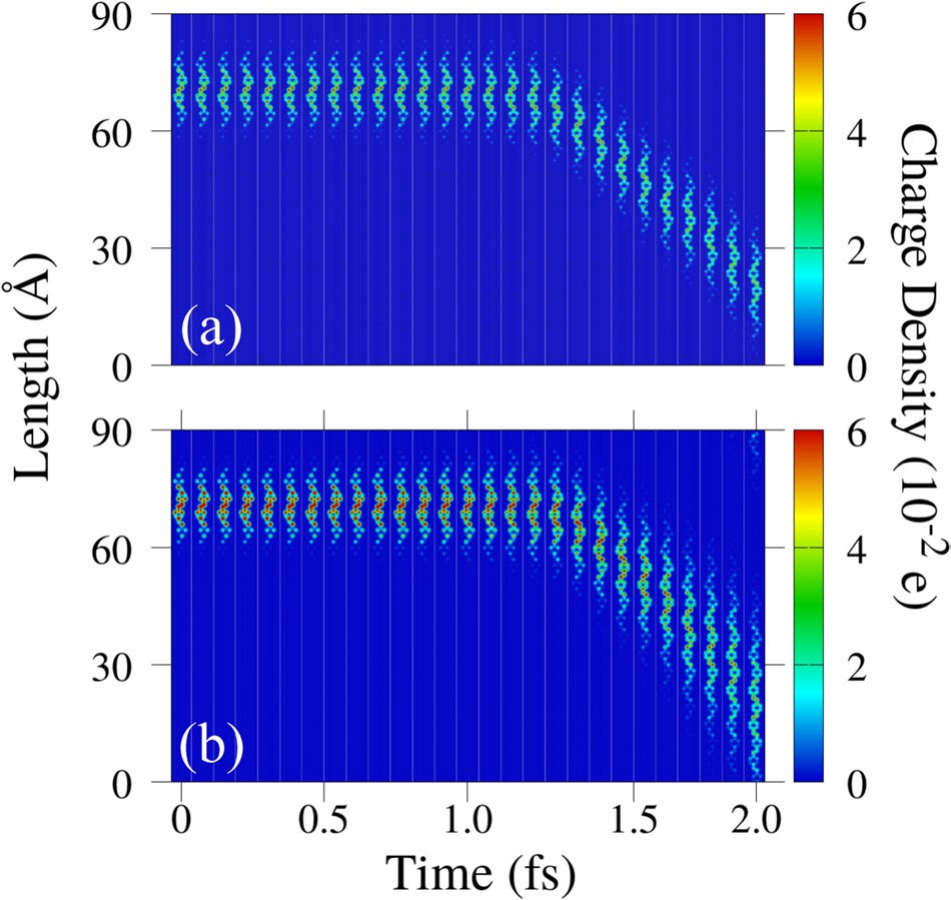
Time evolution of the mean charge density for a PPV lattice containing (**a**) a polaron and (**b**) a bipolaron.

Now we discuss the remarkable transport mechanism of polaron and bipolarons in a PPV chain. Importantly, we note that this process, as discussed below, takes place in this class of materials exclusively. To the best of our knowledge, the transport of these charge carriers in other conjugated polymers^21–23^ and other species of organic semiconducting materials such as molecular crystals^24,25^ and graphene nanoribbons^26,27^ occurs differently. Figure 2(a) depicts, respectively, the time evolution of the mean charge density for a PPV lattice containing a polaron and a bipolaron. In Fig. 2 the dynamics for both charge carriers takes place during two ps for an electric field strength of 1.0 mV/Å. In this figure, one can note that there is a waiting time, about 1.2 ps, where both charge carriers do not move even with the action of the external electric field. Immediately after this transient time, one can realize the polaron and bipolaron start to move linearly as a response to the applied electric field. During their transport, the polaron keeps its initial localization whereas the bipolaron gets even more delocalized at each time step. At 2 ps, the charge delocalization reaches such a level that the bipolaron is no longer stable. Later, we use the time evolution of the energy levels to characterize this loss of stability for the bipolaron case. Surprisingly, the result presented in Fig. 2(b) suggests that bipolarons are not dynamically stable in PPV lattices.

**Figure 3:**
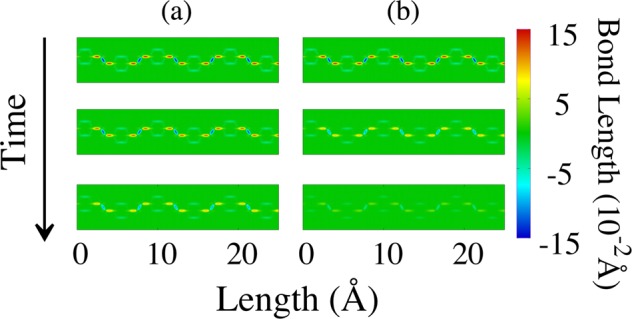
Time evolution of the bond lengths for a PPV lattice containing (**a**–**c**) a polaron and (**d**–**f**) a bipolaron for the cases presented in Fig. 2. Here, we show the three initial time steps for a part of the lattice that does not contains charge (the first 15 Å of its length).

A better understanding of the overall transport mechanism of charge carriers in PPV can be achieved by analyzing the features involved in the time evolution of the bond lengths for the carriers dynamics presented above. Figure 3 illustrates such characteristics in the very few instants of simulation for a part of the lattice that does not contains charge (the first 15 Å of its length). In this figure, it is possible to note that the PPV lattice loses the energy related to the bond conjugation during the time. As a result, the deviations in the bonds, from their equilibrium position, get smaller as the simulation time increases. This process is illustrated here by the lowering in intensity for the red and blue colors that represent, respectively, the *σ* and *π* bonds. We can note that the lattice containing a bipolaron loses much more energy than the one with a polaron. During the process of loss of conjugation, the lattice energy associated with the deviations in the bond lengths is transferred to electrons through the electron-lattice interaction term. Such a mechanism for the energy transference makes the polaron and bipolaron overcome the potential barrier imposed by the local lattice deformations moving as a response to the applied electric field systematically. Since bipolarons have twice more charge than polarons, more lattice energy should be transferred from the lattice to the electronic degree of freedom in the former case to realize the carrier motion. Note that the bond deviations for the bipolaron case almost vanishes. Conversely, the lattice containing a polaron suffers a small change in intensity of the bond deviations but the bond conjugation pattern is still present in an evident fashion. These results for the dynamical lattice arrangement suggests that, in the bipolaron case, a substantial amount of lattice potential energy is converted into kinetic energy for the electrons that form its structure. In the dynamical process, the bipolaron has gained enough energy to overcome the potential barrier associated with the expanded region of its acoustic deformation and then accelerates to a velocity comparable to the polaron case. However, from that moment, the conversion energy mechanism keeps accelerating the bipolaronic charge that decouples from its local lattice deformations by increasing its delocalization. As a consequence, the bipolaron loses its stability. As can be inferred by analyzing both Figs. 2 and 3, the conversion energy mechanism favors the polaron stability and transport. Therefore, these results suggest that the quasiparticles responsible for playing a role in the charge transport in PPV lattices are polarons.

**Figure 4:**
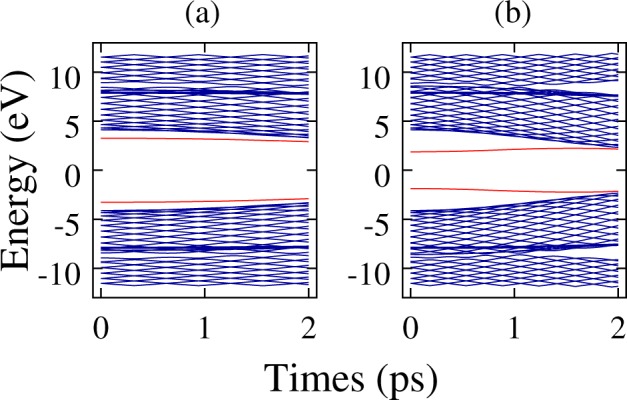
Time evolution of the (**a**) polaron and (**b**) bipolaron energy levels for the cases presented in Fig. 2.

As mentioned above, the dynamical signature for the system’s energy levels helps to better characterize how stable are the charge carriers in a PPV lattice. Finally, we present in Fig. 4(a,b), respectively, the energy levels time evolution for the dynamical simulations of a polaron and a bipolaron shown in Fig. 2. As illustrates Fig. 4, the two intragap levels inside the bandgap at the beginning of the simulation denote the formation of a stable charge carrier. Since a bipolaron deforms much more the lattice than polarons, its energy spectrum can be identified by a couple of states deeper inside the gap when compared to those of a polaron. In Fig. 4(a) one can note that two intragap levels remain consistently inside the bandgap through the rest of the simulation. This result corroborates with the other findings discussed above indicating that the polaron is dynamical stable under the conditions established in the simulation for this particular case. On the other hand, Fig. 4(b) shows the signature of the loss of stability of the bipolaron that is related to the intragap levels that return to the conducting and valence bands.

## Methods

The model Hamiltonian employed here is given by $H={H}_{latt}+{H}_{elec}$, where the first and second terms govern the lattice and electronic degrees of freedom, respectively. By employing a harmonic approximation^28^, we treat the lattice dynamics classically. To avoid edge effects, we consider periodic boundary conditions. In this sense, its Hamiltonian assume the following form

1 $${H}_{latt}=\frac{1}{2}\,\sum _{i}\,\frac{{P}_{i}^{2}}{M}+\frac{1}{2}\,\sum _{\langle i,j\rangle }\,K{\eta }_{i,j}^{2},$$

where *P*_*i*_ is the momentum of the *i*-th site with mass *M*, and *K* is the force constant associated with the *σ* bond^28^.

The electronic Hamiltonian, in turn, describes the *π*-electrons dynamics according to the equation below,

2 $${H}_{elec}=-\,\sum _{\langle i,j\rangle ,s}\,{t}_{i,j}\{{e}^{-i\gamma \overrightarrow{A}\cdot {\hat{r}}_{i,j}}{C}_{i,s}^{\dagger }\,{C}_{j,s}+{e}^{i\gamma \overrightarrow{A}\cdot {\hat{r}}_{i,j}}{C}_{j,s}^{\dagger }\,{C}_{i,s}\}.$$

**Figure 5:**
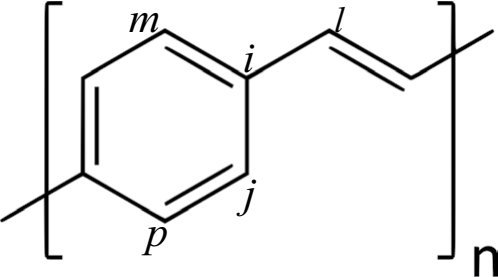
Schematic representation of a PPV lattice.

The summation runs over *π*-electrons in neighboring *i* and *j* sites with spin *s* (see Fig. 5). ${C}_{i,s}^{\dagger }$ and *C*_*i*,*s*_ denote the creation and annihilation of an electron in states denoted by their subscript indices. To consider an external electric field ($\overrightarrow{E}$), we use a vector potential according to $\overrightarrow{E}(t)=-\,(1/c)\dot{\overrightarrow{A}}(t)$. The exponentials come from the Peierls substitution method^29^. The unit vector ${\hat{r}}_{i,j}$ points from *j* site to *i* site. Finally, the parameter *γ* in *H*_*elec*_ is defined as $\gamma \equiv \frac{ea}{\hslash }c$, where *a* is the lattice parameter, *e* the fundamental charge, and *c* the speed of light. The term *t*_*i*,*j*_ is the hopping integral, which couples the *π*-electrons to the lattice according to

3 $${t}_{i,j}={t}_{0}-\alpha {\eta }_{i,j}.$$

In Eq. 3, *α* is the electron-phonon coupling constant and ${\eta }_{i,j}$ is the relative displacement of the lattice sites from their equilibrium positions.

The dynamics calculation starts from an arbitrary initial set of coordinates $\{{\eta }_{i,j}\}$, that is necessary to solve the electronic part of our model Hamiltonian initially. As a consequence, this procedure leads to an eigenvalue-eigenvector equation for the electronic component of the system, where the eigenvalues are *E*_*k*_ and the eigenvectors are ${\psi }_{k,s}(i,t=0)$. These quantities can be related as follows:

4 $${E}_{k}{\psi }_{k,s}(i,t=0)=-\,{t}_{i,j}{\psi }_{l,s}(j,t=0)-{t}_{i,j}{\psi }_{l,s}(j^{\prime} ,t=0)-{t}_{i,j}{\psi }_{l,s}(j^{\prime\prime} ,t=0),$$

where *i*, *j*, *j*′ and *j*″ are neighboring sites.

To solve the classical component or our model, that describes the lattice structure, we turn to the Euler-Lagrange equation^30^. From the solution of the electronic part, we evaluate the expectation value of the wave function $\langle \Psi |L|\Psi \rangle$. This equation leads to:

5 $$\langle L\rangle =\frac{1}{2}\,\sum _{i}\,\frac{{P}_{i}^{2}}{M}-\frac{1}{2}\,\sum _{\langle i,j\rangle }\,K{\eta }_{i,j}^{2}+\sum _{\langle i,j\rangle ,s}\,\{{t}_{i,j}{B}_{i,j}+{\rm{c}}.{\rm{c}}.\},$$

where

6 $${B}_{i,j}=\sum _{k,s}^{\prime} \,{e}^{-i\gamma \overrightarrow{A}\cdot {\hat{r}}_{i,j}}{\psi }_{k,s}^{\ast }(i,t){\psi }_{k,s}(j,t),$$

couples the electronic and lattice degrees of freedom. The primed sum means that only the occupied states are considered.

The solution of the Euler-Lagrange equation with ${P}_{i}=0$ leads to a new set of coordinates $\{{\eta }_{i,j}\}$ that is used to recalculate the electronic Hamiltonian. This process is repeated iteratively until they reach the convergence criteria. As a result, this self-consistent procedure yields the ground state geometry that considers the interdependence between charge and lattice.

After achieving the convergence criteria, the time evolution of the initial state can be accomplished using the full Euler-Lagrange equation^30^. The time evolution of the electronic part is governed employing the time-dependent Schrödinger equation. To do so, we expand the wave function |${\psi }_{k,s}(t)$⟩ in the basis of eigenstates of the electronic Hamiltonian, {|${\phi }_{l,s}(t)$⟩}, at a given time *t*. Therefore, the wave function in time $t+dt$ can be expressed as

7 $$\begin{array}{rcl}|{\psi }_{k,s}(t+dt)\rangle  & = & {e}^{-\frac{i}{\hslash }{\int }_{t}^{t+dt}dt^{\prime} H(t^{\prime} )}|{\psi }_{k,s}(t)\rangle \\  & = & {e}^{-\frac{i}{\hslash }H(t)dt}\,\sum _{l}\,|\phi {(t)}_{l,s}\rangle \langle \phi {(t)}_{l,s}|{\psi }_{k,s}(t)\rangle \\  & = & \sum _{l}\,\langle \phi {(t)}_{l,s}|{\psi }_{k,s}(t)\rangle {e}^{-\frac{i}{\hslash }{\varepsilon }_{l}(t)dt}|\phi {(t)}_{l,s}\rangle ,\end{array}$$

or in terms of eigenfunctions,

8 $${\psi }_{k,s}(i,t+dt)=\sum _{l,m}\,{\phi }_{l,s}(m,t)\,{\psi }_{k,s}(m,t){e}^{(-\frac{{\varepsilon }_{l}(t)}{\hslash }dt)}{\phi }_{l,s}(i,t),$$

where ${\varepsilon }_{l}(t)$ is the eigenenergy of |${\phi }_{l,s}(t)$⟩. The dynamics of the electronic structure is carried out by using Eq. 7, that is evaluated numerically and then employed to the calculation of the expectation value of a new Lagrangian^30^. The Euler-Lagrange equation leads to a Newtonian type expression that takes into account the neighboring bonds:

9 $$\begin{array}{lllll}{F}_{i,j}(t) & = & M{\ddot{y}}_{i,j} & = & \frac{1}{2}K\{{y}_{i,l}+{y}_{m,i}+{y}_{j,p}+{y}_{q,j}-4{B}_{i,j}\}\\  &  &  &  & +\,\frac{1}{2}\alpha \{{B}_{i,l}+{B}_{m,i}+{B}_{j,p}+{B}_{q,j}-4{B}_{i,j}+c.c.\}.\end{array}$$

## Conclusions

In summary, the origin of the charge carrier dynamics in PPV chains under the influence of an external electric field were analyzed employing a 2D tight-binding Hamiltonian that takes into account lattice relaxation effects. Interestingly, our results revealed that the PPV lattice loses the energy related to its conjugation during time by transferring this amount of energy to electrons that yield a polaron and a bipolaron. This energy transference activates their electric field assisted transport. In the bipolaron case, a substantial amount of lattice potential energy is converted into kinetic energy for the electrons that form its structure. In the dynamical process, the bipolaron gains enough energy to overcome the potential barrier associated with the expanded region of its acoustic deformation and then move linearly during 500 fs. However, from that moment, the conversion energy mechanism keeps accelerating the bipolaronic charge that decouples from its local lattice deformations by increasing its delocalization. As a consequence, the bipolaron loses its stability. Conversely, this mechanism for conversion energy favors the polaron stability and transport. Therefore, these results suggest that the quasiparticles responsible for playing a role in the charge transport in PPV lattices are polarons.

## References

[1] Niu Q, Rohloff R, Wetzelaer G-JAH, Blom PWM, Craciun NI (2018). Hole trap formation in polymer light-emitting diodes under current stress. Nat. Mat..

[2] Zhao B (2018). High-efficiency perovskite–polymer bulk heterostructure light-emitting diodes. Nat. Phot..

[3] Friend RH (1999). Electroluminescence in conjugated polymers. Nature.

[4] Heeger Alan J. (2001). Semiconducting and Metallic Polymers: The Fourth Generation of Polymeric Materials (Nobel Lecture). Angewandte Chemie International Edition.

[5] Bredas JL, Street GB (1985). Polarons, bipolarons, and solitons in conducting polymers. Accounts Chem. Res..

[6] Blom PWM, de Jong MJM, Vleggaar JJM (1996). Electron and hole transport in poly(p-phenylene vinylene) devices. Appl. Phys. Lett..

[7] Blom P, Vissenberg M (2000). Charge transport in poly(p-phenylene vinylene) light-emitting diodes. Mater. Sci. Eng. R: Reports.

[8] Blom PWM, de Jong MJM, van Munster MG (1997). Electric-field and temperature dependence of the hole mobility in poly(p-phenylene vinylene). Phys. Rev. B.

[9] Blom PWM, Vissenberg MCJM (1998). Dispersive hole transport in poly(p-phenylene vinylene). Phys. Rev. Lett..

[10] Hulea IN (2004). Wide energy-window view on the density of states and hole mobility in poly(p-phenylene vinylene). Phys. Rev. Lett..

[11] Katsouras I (2013). Charge transport in poly(p-phenylene vinylene) at low temperature and high electric field. Org. Electron..

[12] Markov DE, Tanase C, Blom PWM, Wildeman J (2005). Simultaneous enhancement of charge transport and exciton diffusion in poly(p-phenylene vinylene) derivatives. Phys. Rev. B.

[13] Martens HCF, Blom PWM, Schoo HFM (2000). Comparative study of hole transport in poly(p-phenylene vinylene) derivatives. Phys. Rev. B.

[14] Mihailetchi VD, Wildeman J, Blom PWM (2005). Space-charge limited photocurrent. Phys. Rev. Lett..

[15] Tanase C, Blom PWM, de Leeuw DM (2004). Origin of the enhanced space-charge-limited current in poly(p-phenylene vinylene). Phys. Rev. B.

[16] Hoofman RJOM, de Haas MP, Siebbeles LDA, Warman JM (1998). Highly mobile electrons and holes on isolated chains of the semiconducting polymer poly(phenylene vinylene). Nature.

[17] Martens HCF, Brom HB, Blom PWM (1999). Frequency-dependent electrical response of holes in poly(p-phenylene vinylene). Phys. Rev. B.

[18] Inigo A. R., Tan C. H., Fann W., Huang Y.-S., Perng G.-Y., Chen S.-A. (2001). Non-dispersive Hole Transport in a Soluble Poly(p-phenylene vinylene). Advanced Materials.

[19] Grozema FC, van Duijnen PT, Berlin YA, Ratner MA, Siebbeles LDA (2002). Intramolecular charge transport along isolated chains of conjugated polymers: Effect of torsional disorder and polymerization defects. The J. Phys. Chem. B.

[20] Hultell M, Stafström S (2009). Impact of ring torsion dynamics on intrachain charge transport in conjugated polymers. Phys. Rev. B.

[21] Johansson AA, Stafström S (2004). Nonadiabatic simulations of polaron dynamics. Phys. Rev. B.

[22] Johansson A, Stafström S (2001). Polaron dynamics in a system of coupled conjugated polymer chains. Phys. Rev. Lett..

[23] Ribeiro LA, da Cunha WF, de Oliveria Neto PH, Gargano R, e Silva GM (2013). Effects of temperature and electric field induced phase transitions on the dynamics of polarons and bipolarons. New J. Chem..

[24] Mozafari E, Stafström S (2013). Polaron dynamics in a two-dimensional holstein-peierls system. The J. Chem. Phys..

[25] Junior LAR, Stafström S (2015). Polaron stability in molecular semiconductors: Theoretical insight into the impact of the temperature, electric field and the system dimensionality. Phys. Chem. Chem. Phys..

[26] Ribeiro LA, da Cunha WF, Fonseca ALdA, e Silva GM, Stafstrom S (2015). Transport of polarons in graphene nanoribbons. The J. Phys. Chem. Lett..

[27] de Oliveira Neto PH, Teixeira JF, da Cunha WF, Gargano R, e Silva GM (2012). Electron–lattice coupling in armchair graphene nanoribbons. The J. Phys. Chem. Lett..

[28] Su WP, Schrieffer JR, Heeger AJ (1980). Solitons excitations in polyeacetylene. Phys. Rev. B..

[29] Castro Neto AH, Guinea F, Peres NMR, Novoselov KS, Geim AK (2009). The electronic properties of graphene. Rev. Mod. Phys..

[30] Ferreira da Cunha W, de Oliveira Neto PH, Terai A, Magela e Silva G (2016). Dynamics of charge carriers on hexagonal nanoribbons with vacancy defects. Phys. Rev. B.

